# MS-stable/TMB-high pleomorphic liposarcoma successfully treated with pembrolizumab: a case report

**DOI:** 10.3389/fonc.2025.1552411

**Published:** 2025-06-03

**Authors:** Satoshi Miwa, Hiroshi Kobayashi, Toshihide Hirai, Koichi Okajima, Yuki Ishibashi, Yusuke Tsuda, Liuzhe Zhang, Toshihiko Ando, Aya Shinozaki-Ushiku, Sakae Tanaka

**Affiliations:** ^1^ Department of Musculoskeletal Oncology, Tokyo Metropolitan Cancer and Infectious Diseases Center Komagome Hospital, Tokyo, Japan; ^2^ Department of Orthopaedic Surgery, Faculty of Medicine, The University of Tokyo, Tokyo, Japan; ^3^ Department of Pathology, The University of Tokyo Hospital, Tokyo, Japan

**Keywords:** pleomorphic liposarcoma, tumor mutation burden (TMB), immune checkpoint inhibitors (ICIs), pembrolizumab, liver metastasis

## Abstract

Pleomorphic liposarcoma (PLPS) is a rare and aggressive subtype of liposarcoma with limited treatment options. Despite studies on immune checkpoint inhibitors (ICIs) in sarcomas, there have been few reports involving PLPS. Furthermore, the significance of tumor mutation burden (TMB)-high, a known biomarker for ICIs in various solid tumors, remains unclear in sarcomas. Herein, we report the case of a 41-year-old man with postoperative liver metastasis of microsatellite (MS)-stable/TMB-high PLPS who achieved successful remission with pembrolizumab, an anti-programmed cell death protein 1 inhibitor. Initially, the patient underwent extensive resection for primary PLPS in the distal left thigh. The diagnosis of lung metastases 3 months after prompted five courses of doxorubicin and ifosfamide, resulting in stable disease. Subsequent thoracoscopic pulmonary metastasectomy allowed surgical removal of the lung metastases. However, multiple liver metastases developed 9 months following the primary extensive resection. Cancer genome profiling revealed a mutation in *MSH6*, MS-stable status, and a high TMB of 14.5/Mb. Pembrolizumab was initiated for a total of 35 courses at 10 months postoperatively, significantly reducing liver metastases. These findings suggest the potential of TMB-high as a predictor of ICI response in sarcomas.

## 1 Introduction

Pleomorphic liposarcoma (PLPS) is a rare and aggressive subtype of liposarcoma, accounting for 5–10% of liposarcoma cases, with an overall 5-year survival rate of 54–57% (1, 2). Distant metastases occur in approximately 30–50% of patients, primarily to the lungs, and frequently exhibit limited response to chemotherapy and radiotherapy. Surgical resection with clear margins remains the cornerstone treatment for localized disease. In advanced settings, chemotherapy regimens involving doxorubicin and gemcitabine, along with newer agents such as eliburin and trabectedin, have been utilized (2).

The emergence of immune checkpoint inhibitors (ICIs), such as the anti-programmed cell death protein 1 inhibitor pembrolizumab, has revolutionized treatment algorithms for various malignancies. In contrast, the efficacy of pembrolizumab in sarcomas, particularly PLPS, remains limited. In the SARC028 study, only 18% (7/40 patients) of the patients with specific soft tissue sarcomas (undifferentiated pleomorphic sarcoma, dedifferentiated liposarcoma, and synovial sarcoma) responded to pembrolizumab monotherapy (3). Findings in the SARC028 expansion cohorts demonstrate a limited overall response rate of 10% (4/39 patients) in the liposarcoma cohort (4). On the contrary, a recent randomized control trial has demonstrated that the addition of pembrolizumab to the preoperative radiotherapy and surgery led to a 15% increase in disease-free survival for patients with stage III undifferentiated pleomorphic sarcoma and liposarcoma, indicating the potential of the drug as a promising adjuvant/neoadjuvant therapy (5).

Microsatellite instability (MSI)-high and tumor mutation burden (TMB)-high are established biomarkers for ICIs in various solid tumors (6). However, the utility of these biomarkers for predicting response in sarcomas remains unknown. Therefore, this report describes a case of postoperative liver metastases of MS-stable and TMB-high PLPS that was successfully treated with pembrolizumab.

## 2 Case description

**Figure 1:**
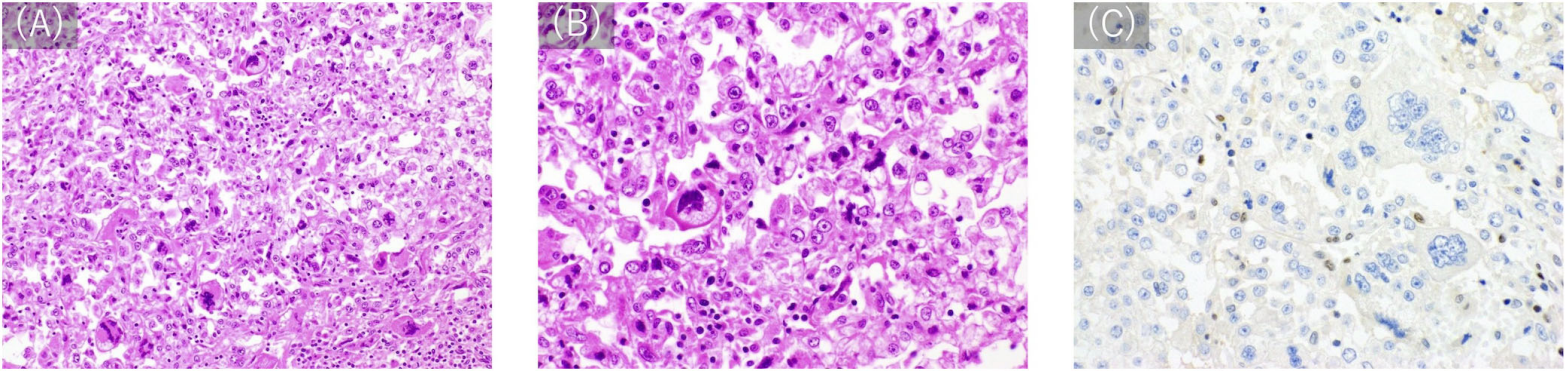
Histological appearance. **(A, B)** Hematoxylin-eosin staining (**A**: 200×, **B** 400×) shows diffuse proliferation of atypical cells with pale to eosinophilic cytoplasm. Some of the tumor cells have vacuole-filled cytoplasms, consistent with the appearance of lipoblasts. Cells show variations from multi-lobed, spindle-shaped cells to those with high N/C ratios and round shapes with malformed and large nuclei. **(C)** Immunostaining with anti-MSH6 (200×) reveals a loss of MSH6 expression.

A 41-year-old man with no significant medical or family history of malignancy presented with a soft tissue tumor in the distal medial left thigh, which was confirmed to be pleomorphic sarcoma following needle biopsy. On physical examination, a 4-cm elastic hard mass was palpable in the distal medial thigh with weight-bearing pain, although, no limitation in the range of motion of the knee joint was observed. Baseline blood tests at the initial visit showed a slightly elevated C-reactive protein (CRP) level of 0.58 mg/dL, while all other parameters, including white blood cell count (WBC), hemoglobin, platelets, renal and liver function markers, and electrolytes, were within normal limits. Subsequent extensive resection with negative surgical margins was performed. Histopathological examination established the diagnosis of PLPS, characterized by the presence of pleomorphic cells with pale to eosinophilic cytoplasm and lipoblasts (**Figures 1A, B**).

**Figure 2:**
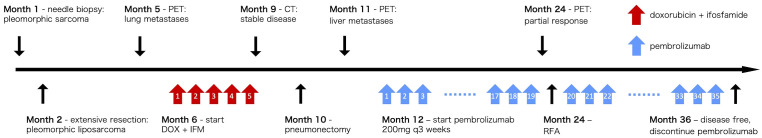
Timeline of significant clinical events and treatment.

**Figure 3:**
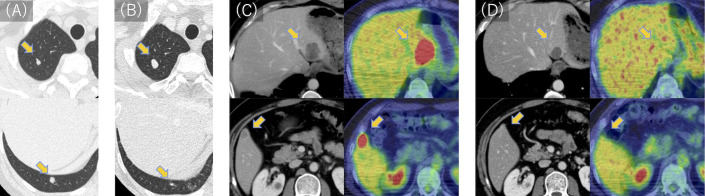
CT and PET scan images of lung and liver metastases during treatment. **(A)** Two lung metastases were found 3 months after wide resection. **(B)** Lung metastases were deemed stable following five courses of doxorubicin and ifosfamide. **(C)** Two liver metastases were identified in the left and right lobes 9 months after wide resection. **(D)** Following 19 courses of pembrolizumab, liver metastases were significantly reduced in the left lobe and became undetectable in the right lobe. Fluorodeoxyglucose uptake was reduced to physiological levels. PET, positron emission tomography; CT, computed tomography.

Three months later, as shown in the clinical course summarized in **Figure 2**, multiple lung metastases were detected (**Figure 3A**), measuring up to 0.7 cm in diameter, necessitating five courses of doxorubicin and ifosfamide. The chemotherapy regimen consisted of doxorubicin at 30 mg/m² and ifosfamide at 2 g/m², without dose reduction, and was completed in five cycles. The patient remained asymptomatic with no respiratory symptoms during the course of pulmonary metastases. After achieving stable disease (**Figure 3B**), thoracoscopic pulmonary metastasectomy was performed to remove the lung metastases. Approximately 9 months following the initial resection, two liver metastatic lesions were identified (**Figure 3C**), measuring 1.5 cm in the right lobe and 4.0 cm in the left lobe. The patient exhibited no symptoms, and liver function tests showed all parameters within the normal range.

**Figure 4:**
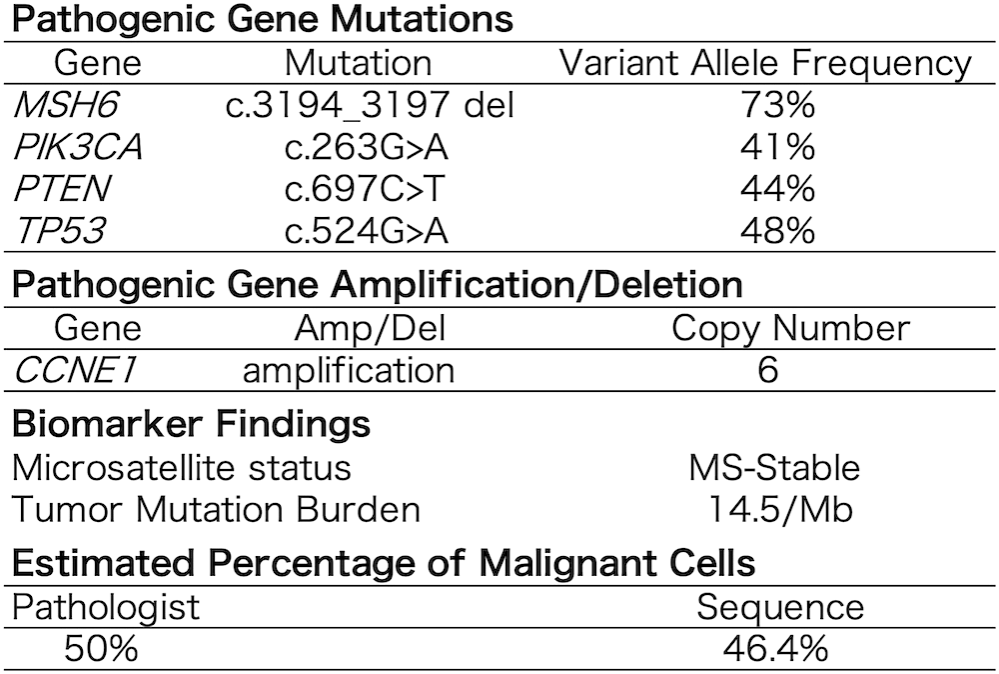
Genomic findings and biomarker findings reported by FoundationOne CDx.

A cancer genome profiling (FoundationOne CDx) of the primary tumor (**Figure 4**) revealed pathogenic gene mutations in *MSH6*, *PIK3CA*, *PTEN*, 1and *TP53* and pathogenic gene amplification of *CCNE1*. The tumor was MS-stable and TMB-high (14.5/Mb). The high mutant allele frequency of *MSH6* (73%) suggested a potential germline mutation, considering the estimated tumor cell content of 46.4–50%. Moreover, immunostaining demonstrated a loss of MSH6 expression (**Figure 1C**).

Given these findings, pembrolizumab 200 mg was initiated at 10 months postoperatively, which was administered every 3 weeks. Before initiation, serum amylase and thyroid function tests (TSH, FT3, FT4) were within normal limits. While the patient experienced thyrotoxicosis and hyperamylasemia during treatment, both of them were asymptomatic. TSH level decreased to 0.01 μIU/mL after two cycles but normalized the following month without intervention. Later, after the 31st cycle, TSH elevation was observed (Grade 1); pembrolizumab was continued. Subsequent to the 32nd cycle, TSH rose to 22.58 μIU/mL, with FT3 and FT4 remaining normal (Grade 2). The patient remained asymptomatic and was started on levothyroxine replacement. Amylase levels increased to 338 U/L (Grade 3) after approximately one year of treatment, and the patient remined asymptomatic. The levels normalized within three months without intervention.

A significant clinical response was observed, with the liver metastasis in the right lobe completely disappearing and the left lobe lesion reducing from 4.0 cm to 2.5 cm in diameter (**Figure 3D**). Furthermore, positron emission tomography-computed tomography revealed normal physiological levels of fluorodeoxyglucose uptake. Subsequently, radiofrequency ablation (RFA) was performed on the residual lesion in the left lobe. Pembrolizumab was continued as initially planned, and the patient completed 35 cycles over a 2-year period. As of the latest follow-up, the patient remains disease-free, and pembrolizumab has been discontinued. The patient is now under surveillance without active treatment.

## 3 Discussion

We report a case of postoperative liver metastasis from an MS-stable and TMB-high PLPS that was successfully treated with pembrolizumab, implying the utility of TMB-high as a biomarker for ICI response in sarcomas. To the best of our knowledge, this is the first report on the efficacy of pembrolizumab in PLPS with TMB-high.

TMB-high, a well-established biomarker for predicting response to ICIs in solid tumors, may demonstrate similar value in sarcomas, as suggested in our present case. The KEYNOTE-158 study reported the potential benefit of ICIs in TMB-high (≥10 mutations/Mb) solid tumors, regardless of MSI. While their findings expanded the number of candidates eligible for ICIs, patients with sarcoma were excluded from the cohort (7). Another study involving 60 cases, including three cases of sarcoma, showed that MS-stable and TMB-high (≥20 mutations/Mb) tumors could benefit from immunotherapy (8). Additional case reports of undifferentiated pleomorphic sarcoma, alveolar soft part sarcoma, intimal sarcoma, and osteosarcoma also support the association between TMB-high status and ICI response in sarcomas (9, 10). However, none of them included PLPS. Given the significantly higher prevalence of TMB-high (≥20 mutations/Mb) sarcomas (11) (8.1%) than MSI-high sarcomas (0.26–0.29%) (12, 13), further investigation into the utility of TMB-high status as a predictive biomarker in sarcomas is warranted to identify parients who can benefit from ICIs.

Similarly, the significance of mutations in the DNA mismatch repair (MMR) genes on the therapeutic effect of pembrolizumab in MS-stable/TMB-high malignancies remains unknown. Although other prognostic biomarkers for ICIs in sarcoma are less established, recent reports have focused on the tumor microenvironment. The SARC028 sub-analysis demonstrated that B cell infiltration and tertiary lymphoid structures (TLSs) were key predictors of improved survival, proposing the sarcoma immune class classification as a biomarker of pembrolizumab efficacy (14). The PEMBROSARC trial further explored the role of TLSs, correlating TLS positivity with improved outcomes and increased responsiveness to combined pembrolizumab and low-dose cyclophosphamide (15). Additionally, the efficacy of pembrolizumab has been reported in alveolar soft part sarcoma (16), characterized by low TMB, a specific fusion gene, and an inactive immune microenvironment. This suggests that a comprehensive understanding of sarcomas as a whole, including microenvironment, gene alterations, and histologic type, is crucial in identifying patients who may benefit from ICIs.

The development of this MS-stable and TMB-high tumor in this patient may be attributed to a germline mutation in *MSH6*. In Lynch syndrome (LS), germline mutations in MMR genes (*MLH1*, *MSH2*, *MSH6*, *PMS2*, and *EPCAM*) predispose individuals to various malignancies, including colorectal and endometrial cancers. Although sarcomas are not traditionally considered LS-associated cancers, individuals with LS exhibit a higher incidence and an earlier age of onset of sarcomas than the general population (17–19). The distribution of soft tissue sarcomas in patients with LS differs from that of the general population, particularly with regard to the high proportion (10%) of pleomorphic rhabdomyosarcoma, which is rare (<1%) in sporadic soft tissue sarcomas (20). MMR gene deficiency primarily leads to high MS instability owing to impaired gene repair capacity. However, MSH6 mutations generally involve base-base mismatches rather than larger insertion/deletion mismatches (21). Therefore, tumors with *MSH6* mutations can be MS-stable and TMB-high. Indeed, pan-cancer analyses of LS have revealed a significant prevalence of *MSH6* mutations in MS-stable tumors (22).

## 4 Conclusion

We presented a case of MS-stable and TMB-high PLPS that was successfully treated with pembrolizumab. Our findings suggest that TMB-high is a potential predictor of the therapeutic response to ICIs in sarcoma. Further investigations involving more cases are necessary to validate this association.

## Data Availability

The original contributions presented in the study are included in the article/supplementary material. Further inquiries can be directed to the corresponding author.

## References

[1] Lee ATJ Thway K Huang PH Jones RL. Clinical and molecular spectrum of liposarcoma. J Clin Oncol. (2018) 36:151–9. doi: 10.1200/JCO.2017.74.9598 PMC5759315 29220294

[2] Assi T Ngo C Faron M Verret B Lévy A Honoré C. Systemic therapy in advanced pleomorphic liposarcoma: a comprehensive review. Curr Treat Options Oncol. (2023) 24:1598–613. doi: 10.1007/s11864-023-01139-3 37843627

[3] Tawbi HA Burgess M Bolejack V Van Tine BA Schuetze SM Hu J. Pembrolizumab in advanced soft-tissue sarcoma and bone sarcoma (SARC028): a multicentre, two-cohort, single-arm, open-label, phase 2 trial. Lancet Oncol. (2017) 18:1493–501. doi: 10.1016/S1470-2045(17)30624-1 PMC7939029 28988646

[4] Burgess MA Bolejack V Schuetze S Van Tine BA Attia S Riedel RF. Clinical activity of pembrolizumab (P) in undifferentiated pleomorphic sarcoma (UPS) and dedifferentiated/pleomorphic liposarcoma (LPS): final results of SARC028 expansion cohorts. J Clin Oncol. (2019) 37:11015–5. doi: 10.1200/JCO.2019.37.15_suppl.11015

[5] Mowery YM Ballman KV Hong AM Schuetze SM Wagner AJ Monga V. Safety and efficacy of pembrolizumab, radiation therapy, and surgery versus radiation therapy and surgery for stage III soft tissue sarcoma of the extremity (SU2C-SARC032): an open-label, randomised clinical trial. Lancet. (2024) 404:2053–64. doi: 10.1016/S0140-6736(24)01812-9 PMC11842127 39547252

[6] Li Y Ma Y Wu Z Zeng F Song B Zhang Y. Tumor mutational burden predicting the efficacy of immune checkpoint inhibitors in colorectal cancer: a systematic review and meta-analysis. Front Immunol. (2021) 12:751407. doi: 10.3389/fimmu.2021.751407 34659255 PMC8511407

[7] Marabelle A Fakih M Lopez J Shah M Shapira-Frommer R Nakagawa K. Association of tumour mutational burden with outcomes in patients with advanced solid tumours treated with pembrolizumab: prospective biomarker analysis of the multicohort, open-label, phase 2 KEYNOTE-158 study. Lancet Oncol. (2020) 21:1353–65. doi: 10.1016/S1470-2045(20)30445-9 32919526

[8] Goodman AM Sokol ES Frampton GM Lippman SM Kurzrock R. Microsatellite-stab le tumors with high mutational burden benefit from immunotherapy. Cancer Immunol Res. (2019) 7:1570–3. doi: 10.1158/2326-6066.CIR-19-0149 PMC6774837 31405947

[9] Li Y Liu Y Qu Y Chen X Qu X Ye Y. Case report: two cases of soft-tissue sarcomas: high TMB as a potential predictive biomarker for anlotinib combined with toripalimab therapy. Front Immunol. (2022) 13:832593. doi: 10.3389/fimmu.2022.832593 35603147 PMC9120574

[10] Shehata MS Lofftus SY Park JY Singh AS Federman NC Eilber FC. Sarcoma in patients with Lynch syndrome and response to immunotherapy. J Surg Oncol. (2024) 129:820–6. doi: 10.1002/jso.27567 38151827

[11] Chalmers ZR Connelly CF Fabrizio D Gay L Ali SM Ennis R. Analysis of 100,000 human cancer genomes reveals the landscape of tumor mutational burden. Genome Med. (2017) 9:34. doi: 10.1186/s13073-017-0424-2 28420421 PMC5395719

[12] Gounder MM Agaram NP Trabucco SE Robinson V Ferraro RA Millis SZ. Clinical genomic profiling in the management of patients with soft tissue and bone sarcoma. Nat Commun. (2022) 13:3406. doi: 10.1038/s41467-022-30496-0 35705558 PMC9200814

[13] Nacev BA Sanchez-Vega F Smith SA Antonescu CR Rosenbaum E Shi H. Clinical sequencing of soft tissue and bone sarcomas delineates diverse genomic landscapes and potential therapeutic targets. Nat Commun. (2022) 13:3405. doi: 10.1038/s41467-022-30453-x 35705560 PMC9200818

[14] Petitprez F de Reyniès A Keung EZ Chen TWW Sun CM Calderaro J. B cells are associated with survival and immunotherapy response in sarcoma. Nature. (2020) 577:556–60. doi: 10.1038/s41586-019-1906-8 31942077

[15] Italiano A Bessede A Pulido M Bompas E Piperno-Neumann S Chevreau C. Pembrolizumab in soft-tissue sarcomas with tertiary lymphoid structures: a phase 2 PEMBROSARC trial cohort. Nat Med. (2022) 28:1199–206. doi: 10.1038/s41591-022-01821-3 35618839

[16] Wilky BA Trucco MM Subhawong TK Florou V Park W Kwon D. Axitinib plus pembrolizumab in patients with advanced sarcomas including alveolar soft-part sarcoma: a single-centre, single-arm, phase 2 trial. Lancet Oncol. (2019) 20:837–48. doi: 10.1016/S1470-2045(19)30153-6 31078463

[17] Dominguez-Valentin M Sampson JR Møller P Seppälä TT Plazzer JP Nakken S. Analysis in the Prospective Lynch syndrome Database identifies sarcoma as part of the Lynch syndrome tumor spectrum. Int J Cancer. (2021) 148:512–3. doi: 10.1002/ijc.33214 32783184

[18] Nilbert M Therkildsen C Nissen A Åkerman M Bernstein I. Sarcomas associated with hereditary nonpolyposis colorectal cancer: broad anatomical and morphological spectrum. Fam Cancer. (2009) 8:209–13. doi: 10.1007/s10689-008-9230-8 19130300

[19] Carvalho N BN N VN K FD C de MMP Kupper BEC. Clinical and molecular assessment of patients with Lynch syndrome and sarcomas underpinning the association with MSH2 germline pathogenic variants. Cancers. (2020) 12:1848. doi: 10.3390/cancers12071848 32659967 PMC7408879

[20] Poumeaud F Valentin T Vande Perre P Jaffrelot M Bonnet D Chibon F. Special features of sarcomas developed in patients with Lynch syndrome: a systematic review. Crit Rev Oncol Hematol. (2023) 188:104055. doi: 10.1016/j.critrevonc.2023.104055 37301271

[21] Edelmann W Yang K Umar A Heyer J Lau K Fan K. Mutation in the mismatch repair gene Msh6 causes cancer susceptibility. Cell. (1997) 91:467–77. doi: 10.1016/s0092-8674(00)80433-x 9390556

[22] Latham A Srinivasan P Kemel Y Shia J Bandlamudi C Mandelker D. Microsatellite instability is associated with the presence of Lynch syndrome pan-cancer. J Clin Oncol. (2018) 37:286–95. doi: 10.1200/JCO.18.00283 PMC6553803 30376427

